# The Microstructure and Properties of Ni60/60% WC Wear-Resistant Coatings Prepared by Laser-Directed Energy Deposition

**DOI:** 10.3390/mi15091071

**Published:** 2024-08-25

**Authors:** Husen Yang, Wen Li, Yichun Liu, Fengxian Li, Jianhong Yi, Jürgen Eckert

**Affiliations:** 1 Faculty of Material Science and Engineering, Kunming University of Science and Technology, Kunming 650093, China; wy1282223420@163.com (H.Y.); lw1186637944@163.com (W.L.); spsjtu@163.com (Y.L.); yijianhong@kmust.edu.cn (J.Y.); 2 Erich Schmid Institute of Materials Science, Austrian Academy of Sciences, Jahnstrasse 12, A-8700 Leoben, Austria

**Keywords:** laser-directed energy deposition, Ni60/WC wear-resistant coating, laser power

## Abstract

Ni60/60% WC composite coatings with a good surface roughness and high mechanical properties were successfully prepared on 316L stainless steel substrate by laser-directed energy deposition (LDED) technology. The effects of laser power on the microstructural evolution and mechanical properties of the Ni60/60% WC composite coating were investigated. The relationships between the chemical composition, the microstructure, the hardness, and the friction wear resistance of the composite coatings were characterized and investigated. The results show that the laser power had a significant effect on the energy input, which determined the melting extent of the Ni60 phases around the WC particles and the bonding strength between the reinforcements and the matrix, as well as the bonding strength between the substrate and the coatings. With an increase in the laser power from 800 W to 1400 W, the average hardness of the coating surface increased due to the increased densification of the deposited coatings and then decreased due to grain coarsening under a high energy input. The average coefficient of friction of the coatings decreased gradually to 0.383 at 1000 W, showing a minimum wear of 0.00013 mm^2^ at 1200 W. The main wear mechanisms on the coated surfaces were adhesive wear and abrasive wear. Moreover, the coatings deposited at 1200 W exhibited better forming quality and wear resistance. This work suggests that the processing parameters during LDED can be optimized to prepare Ni60/60% WC wear-resistant coatings with excellent mechanical properties.

## 1. Introduction

Steels parts, which are widely applied in various fields, always suffer from failure problems because of their low wear resistances in extreme service conditions. In order to reduce the wear of steel parts, a prevalent approach involves applying coatings on the surface of these parts to enhance their wear resistance and extend their service time [1].

Currently, the process of preparing wear-resistant coatings mainly includes thermal spraying, laser cladding, and so on. Laser cladding technology exhibits promising prospects in the field of metal surface engineering. It is an emerging technique for producing coatings with stable microstructures and excellent mechanical properties [2]. Laser-directed energy deposition (LDED) as a laser additive manufacturing (AM) technology enables the deposition of coatings with a high accuracy and stable energy input, and the excellent performance of coatings by means of a high-energy laser beam has attracted the attention of many researchers in recent years [3,4]. 

The 316L stainless steel parts used in extreme environments inevitably suffer from localized corrosion problems, including pitting corrosion, crevice corrosion, and stress corrosion cracking, which, in turn, affect their service safety and service life [5]. 

Therefore, it is crucial to develop coatings for 316L stainless steel parts that exhibit high corrosion resistance and wear resistance. Ni60, an alloy powder, renowned for its excellent corrosion resistance, pronounced high-temperature self-lubrication properties, and economical cost, has emerged as a ubiquitous surface coating material, which has remarkable abrasion resistance, excellent heat corrosion resistance, and good heat fatigue resistance.

However, the available literature shows the inadequacy of standalone nickel-based coating materials means that they are no longer well suited to meeting evolving demands. Consequently, the preparation of Ni-based composite coatings has led to a substantial enhancement in the mechanical properties of the coatings. Xu et al. [6] successfully prepared a dense Ni-8 wt% Al (Ni-8Al) coating onto a copper substrate using high-speed laser cladding technology. In addition, the microstructure evolution and microhardness of the coating provided valuable insights; i.e., the incorporation of WC into composite coatings provides a significant improvement in wear resistance and hardness. 

Li et al. [7] reported that the composite coatings significantly enhanced the material surface properties. The varying concentrations of WC had a significant effect on the properties of the WC/Ni60 composite coatings, particularly in terms of their mechanical properties. 

You et al. [8] fabricated WC-reinforced Ti6Al4V alloy coatings using laser melting cladding, and the results showed that the hardness enhancement improved the wear resistance. Yang et al. [9] prepared WC/Ti6Al4V composite coatings by adding WC to Ti6Al4V alloy and found that the coatings still exhibited a good metallurgical bonding strength. Wang et al. [10] investigated nickel-based WC coatings obtained by laser melting on 4Cr14Ni14NW2Mo steel and showed that the refined microstructure of the coatings provided significant resistance to abrasive wear at high temperatures. These studies have shown that, during the laser melting and cladding process, the morphology and mechanical properties of the coating are greatly affected by the laser power parameters [11,12,13]. However, the rapid cooling principle of the laser melting and cladding process is likely to lead to some defects inside the coatings, such as holes and cracks [14,15]. The formation of cracks may be caused by the mismatch of the thermal expansion coefficients [16], which need to be further investigated.

Although extensive research has explored the effects of processing on the coating structure and performance, there has been limited investigation into how laser power affects the formation quality and mechanical properties of Ni60/60% WC composite coatings on a 316L stainless steel substrate. This study utilized the LDED technology to fabricate Ni60/60% WC composite coatings on a 316L stainless steel substrate. The effects of laser power on the microstructural evolution and mechanical properties of the Ni60/60% WC composite coating were investigated. The relationships between the chemical composition, the microstructure, the hardness, and the friction wear resistance of the composite coatings were characterized and investigated. This work suggests that the processing parameters during LDED can be optimized to prepare Ni60/60% WC wear-resistant coatings with excellent mechanical properties. These studies are not only of great significance for the practical application of coatings but also have a certain reference value for follow-up research.

## 2. Materials and Methods

### 2.1. Materials

316L stainless steel with a size of 100 × 100 × 5 mm was selected as the substrate. Ni60 powder and WC powder (Qinghe County Chuangjia Welding Materials Co., Ltd., Xingtai, China) with an average particle size of 45 μm was used for the coating deposition. The chemical compositions of the substrate 316L stainless steel and Ni60 powder are given in Table 1. The mixed Ni60/60% WC powder was ball milled (XQM-2 type produced by Changsha Tianchuang Powder Technology Co., Ltd., Changsha, China) with a ball to material ratio of 1:1 and a rotational speed of 180 r/min for 3 h, while the spherical Ni60 powder had a mass fraction of 40% and the irregular WC powder had a mass fraction of 60%.

**Figure 1:**
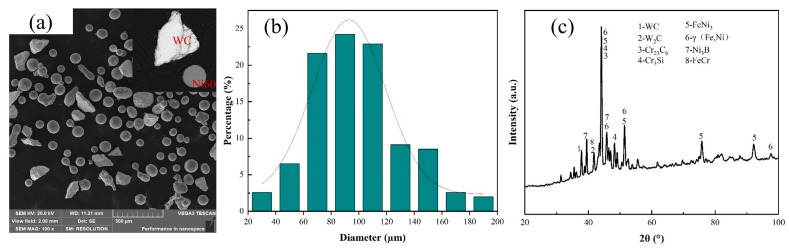
(**a**) SEM image of Ni60/60% WC powder; (**b**) particle size distribution of hybrid powder; and (**c**) XRD image of hybrid powder.

Figure 1a shows the morphological characterization of the mixed powders using a TESCAN VEGA3 scanning electron microscope(TESCAN VEGA3, TESCAN company, Brno, Czech Republic), from which it can be seen that the mixed powder has a good spherical shape, which ensures good flowability in the experiment. Figure 1b exhibits the particle size distribution of the mixed powders. It can be seen that the average size of the composite powder is from 40 to 160 μm. Figure 1c shows the XRD pattern of the mixed composite powders. 

### 2.2. Experimental Method 

**Figure 2:**
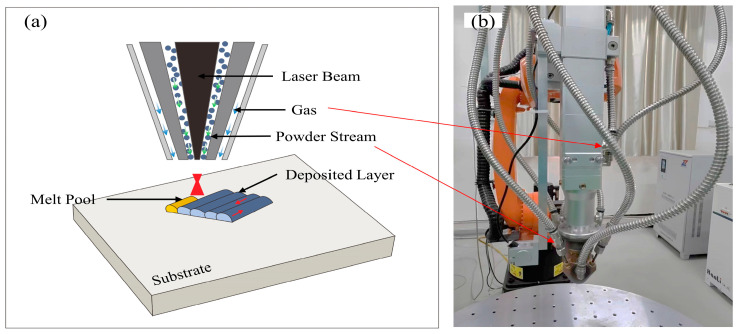
A schematic diagram (**a**) and a photo (**b**) of the printing nozzle when using a powder feeding system.

First, the substrate surface was polished by using 400 #, 600 #, 1000 #, and 2000 # sand paper and then ultrasonic cleaned. By using the LDED system (Digilight-4000, Kunming University of Science and Technology, Kunming, China), as shown in Figure 2b, the mixed powders were filled and delivered to the printing nozzle by using a powder feeding system under an argon atmosphere protection and a gas pressure of 0.5 MPa. The argon used for the protection was 99.999% pure. Then, the powders were deposited on an 316Lsubstrate at a powder flow rate of 10 g/min, as shown in Figure 2a. In order to understand the effects of laser power on the microstructure and mechanical properties of the Ni60/60% WC composite coatings, the processing parameters were designed as shown in Table 2.

The LDED processed coatings were machined into 18 × 18 × 5 mm block samples by EDM wire cutting, after which they were ground and polished to obtain metallographic specimens. The microstructure evolution of the coatings was observed using a Zeiss AXIOScope A1(Axio Scope. A1, Zeiss, Germany) optical microscope and a TESCAN VEGA3 scanning electron microscope equipped with an energy dispersive spectrometer (EDS). Moreover, an X-ray diffractometer (XRD, Mini Flex 600, Rigaku Corporation, Tokyo, Japan) was used for the phase analysis, using a copper target with a tube voltage of 40 kV, a tube current of 300 mA, a scanning speed of 5°/min, and a scanning angle step of 0.01°. The diffraction angle scanning range was from 20° to 100°. The microhardness measurements were carried out using a Zwick microhardness ((HVS-1000Z, Shanghai Yanrun Opto-electro-mechanical Technology Co., Ltd., Shanghai, China) tester at 14 positions equal distances from the top of the coating to the substrate with an applied load of 4.9 N for 15 s. In order to minimize the error, three points at the same height at each distance from the coating position were selected for averaging. The friction wear test was carried out using a multifunctional rotary friction tester (MS-M9000, Lanzhou Huahui Instrument Technology Co., Ltd., Lanzhou, China), while the friction partner was a 5 mm diameter alumina ceramic ball with a high hardness (2000 to 2100 HV); the load was 15 N, the radius of rotation was 5 mm, the loading time was 60 min, and the rotating speed of the rotating disc was 200 r/min. After the experiment, the changes in the wear patterns and the wear areas of the different treated specimens were observed and analyzed.

## 3. Results

### 3.1. Microstructure and Phase Distribution

**Figure 3:**
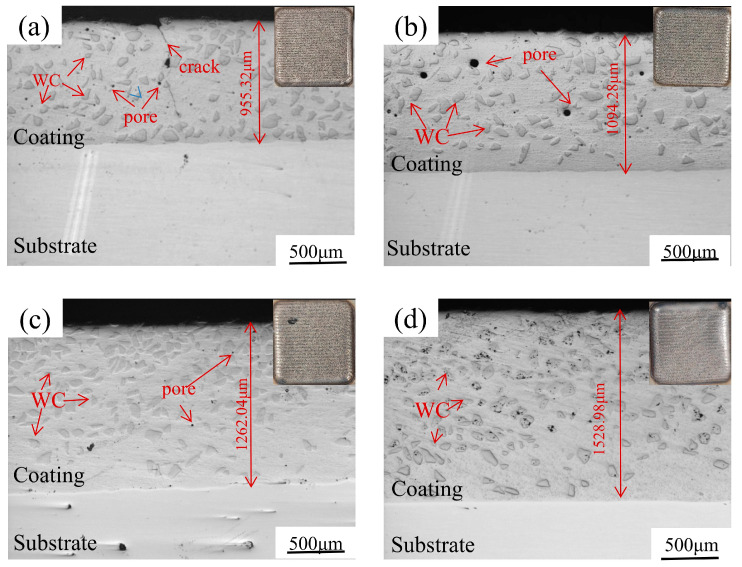
Cross-sectional morphology of the LDED Ni60/60% WC composite coatings deposited at different laser powers: (**a**) 800 W; (**b**) 1000 W; (**c**) 1200 W; and (**d**) 1400 W.

Figure 3 shows the cross-sectional microstructure and surface morphology of the Ni60/60% WC coatings deposited by LDED at different laser powers. As can be seen from Figure 3a, at a laser power of 800 W, the surface morphology of the fusion-coated layer is well molded and there are some unmelted powder particles on the surface, but the fusion between the channels is good, and there are no obvious cracks. As the laser power increases to 1000 W, as shown in Figure 3b, it can be found that the surface of the coating becomes smoother and the thickness of the fused coating layer increases to 1094.28 μm. Although a few defects such as cracks and pores appeared inside the fused cladding layer at 800 W, the cracks and pores disappeared in the deposited coatings when the laser power was larger than 1000 W, as shown in Figure 3c,d.

**Figure 4:**
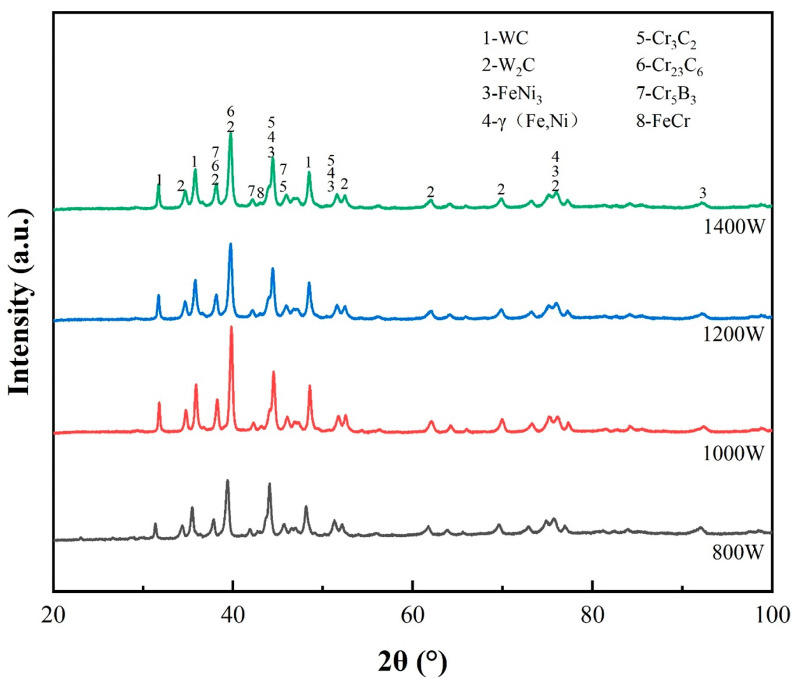
XRD diffraction patterns of the LDED coatings deposited at different laser powers.

Figure 4 shows the XRD diffraction patterns of the LDED coatings deposited at different laser powers. It can be seen that the phases mainly include WC, FeNi_3_, Cr_3_C_2_, Cr_23_C_6_, Cr_5_B_3_, γ-(Fe, Ni), FeCr, and W_2_ C. For the WC particle-reinforced Ni60 composite coatings, γ- (Fe, Ni) acts as the binder metal matrix phase, while W_2_ C, Cr _3_C_2_, and Cr_23_C_6_ act as the reinforcement phases. These phases played a significant role in enhancing the wear resistance and hardness of the materials.

### 3.2. Microhardness and Wear Resistance

**Figure 5:**
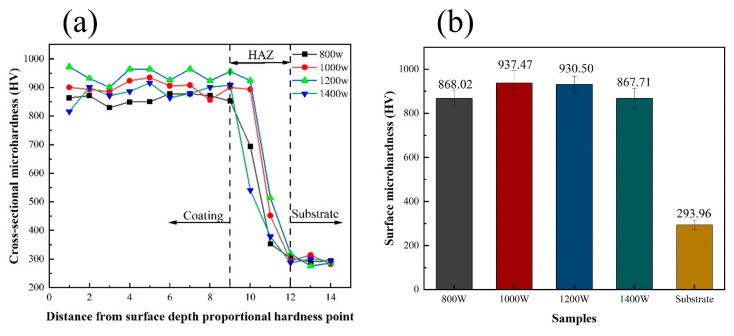
(**a**) Microhardness distribution of the LDED coatings from the coating surface to the substrate under different laser powers, and (**b**) average hardness of the coatings under different powers.

Figure 5a shows the microhardness distribution from the surface of the coating to the substrate for the Ni60/60% WC composite coatings deposited at different laser powers. It can be seen that the microhardness increases relative to the distance between the substrate decreasing and the test point. Moreover, the microhardness in the bonding area between the substrate and the coating increases smoothly. Figure 5b shows that, with an increase in the laser power from 800 W to 1400 W, the average microhardness of the coating surface decreased from 868.02 to 867.71 HV. Furthermore, the values of the microhardness were about three times those of the substrate (293.96 HV). Under appropriate LDED processing parameters, the coatings with a high surface hardness exhibit increased resistance to plastic deformation, thereby mitigating the generation of wear debris and effectively reducing secondary wear [17].

**Figure 6:**
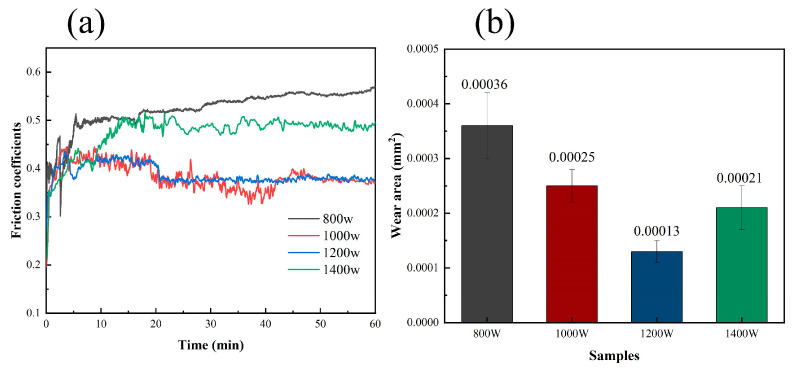
Frictional wear of the LDED coatings deposited at different laser powers: (**a**) friction coefficient; and (**b**) average wear.

Figure 6a shows the friction factor curve of the Ni60/60% WC composite coating. From the results, it can be seen that the friction factor of the composite coating fluctuates greatly from 0.3 to 0.55 in 20 min, which is due to the lack of lubrication between the friction partner and the coating in the unstable friction stage, resulting in high friction in the whole system. Then, the friction factor decreases significantly and tends to change steadily. Figure 6b shows that the wear amount first decreases and then increases with the increase in the laser power. The minimum wear amount of 0.00013 mm^2^ is shown at 1200 W. Since a low laser power can lead to the consequence of incomplete melting of the composite powder, a heightened coefficient of friction arises. Conversely, the coatings that undergo treatment with a high laser power demonstrate a superior resistance to abrasion. However, the application of excessive laser power can result in the generation of internal stresses during LDED, potentially causing the material to crack or peel, thereby adversely impacting its wear performance.

The wear test of the samples was carried out under the same test conditions by tribological testing of the Ni60/60% WC composite coatings at different laser powers. The results show that the proper laser power can improve the wettability of the WC particles in the matrix, improving the wear resistance of the coatings. These coatings slide relative to each other when subjected to shear forces during friction, which mitigates the effects of shear forces on the coatings [18]. When the laser power increases from 800 W to 1400 W, the friction coefficients of the coatings decrease from 0.523 to 0.383, then increase to 0.473.

## 4. Discussion

### 4.1. Microstructure Evolutions

**Figure 7:**
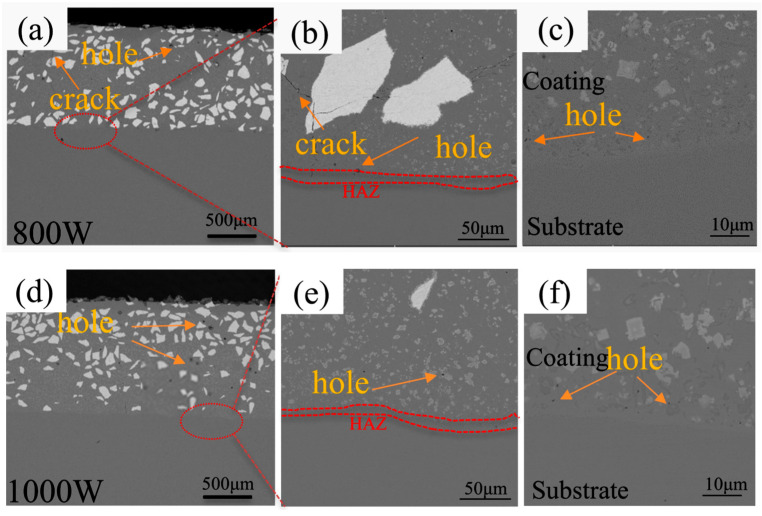

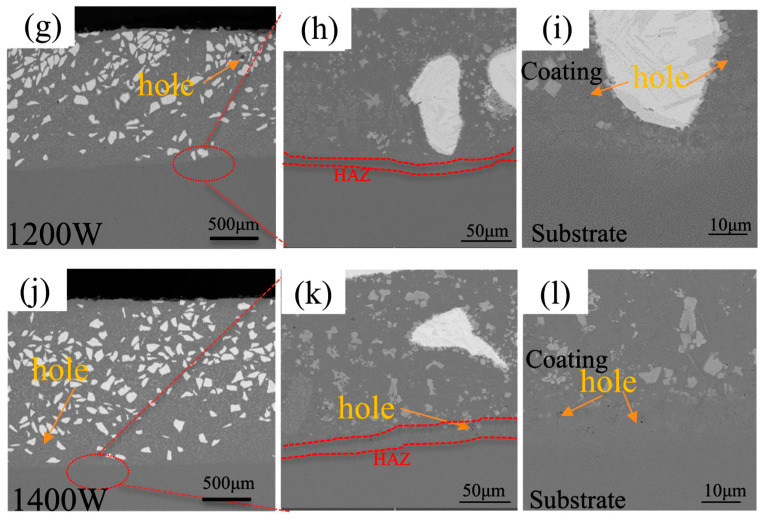
SEM images of the LDED Ni60/60% WC composite coating deposited at different laser powers: 800 W (**a**–**c**); 1000 W (**d**–**f**); 1200 W (**g**–**i**); and 1400 W (**j**–**l**).

Figure 7 shows the SEM images of the Ni60/60% WC composite coatings deposited at different laser powers. A low laser power results in a shallower molten layer and a smaller melting depth, and the rapid cooling of the molten area results in smaller grains. A higher laser power results in a deep melt zone with significant microstructural changes in the melt zone. A higher power causes the grain coarsening. Lots of white unmelted WC phase and grayish-white Cr-W containing fine phases and black substrate metal can be observed in the microstructure of all coatings. WC particles with a high hardness that composite well with the Ni60 can significantly enhance the coating’s hardness, thereby improving its wear resistance. As shown in Figure 7a–c, fine cracks and pores appeared inside the fused cladding layer at 800 W but disappeared when the laser power increased to 1000 W, as shown in Figure 7d–f, indicating a better deposition quality of the fused cladding layer and good metallurgical bonding with the substrate. Figure 7g–i show that the number of pores decreased when the laser power increased to 1200 W, as shown in Figure 7h. However, fine porosity was observed at the interface between the fused cladding and the substrate. The formation of porosity may be related to the entry of protective gas during the rapid cooling and melting process. Porosity can reduce the overall density and strength of a coating. Typically, it acts as a stress concentration point, making the coating more prone to cracking or delamination during frictional processes. The lower laser power is unlikely to melt most of the WC particles, so 800 W is more likely to produce cracks. Figure 7j–l show that, at a laser power of 1400 W, the input energy density is higher, resulting in more unstable melt flow and the formation of more pores. Due to rapid cooling and solidification, a narrower fusion zone appears at the coating–substrate bond.

### 4.2. Strength Mechanisms

The XRD test results show that WC is always present, and this result proves the presence of incompletely melted WC particles in the coating. Under the radiation of a very high energy density laser beam, WC partially dissolves into W and C atoms. C reacts with elements such as Cr to produce other hard phases. As the laser power is gradually increased, the amount of WC particles that are not completely dissolved decreases, and the appearance of the W_2_ C phase indicates that the WC has decomposed, while the W_2_ C phase is in situ generated and W plays a solid solution strengthening role in composite coatings [7]. WC can undergo the following reactions:

(1) $$2WC\to W_{2}C+C$$

(2) $$W_{2}C\to 2W+C$$

**Figure 8:**
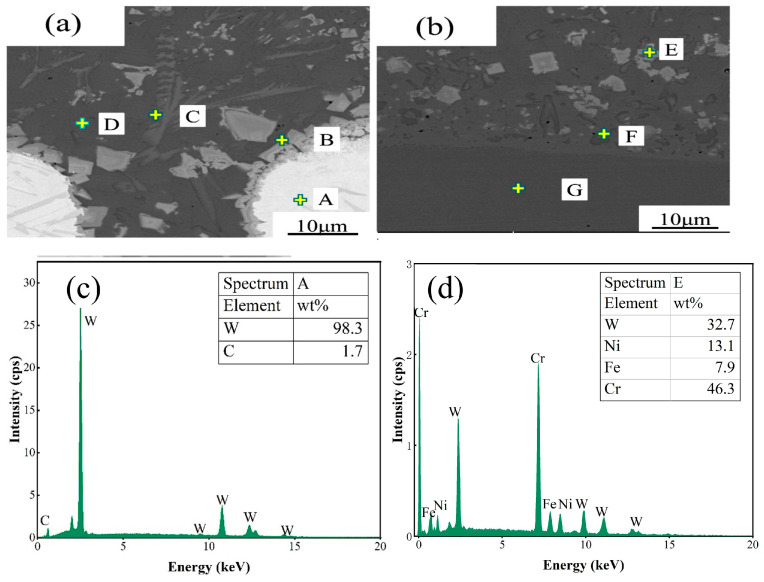
EDS analysis of the LDED Ni60/60% WC composite coating deposited at a laser power of 1200W: (**a**) Scanning points in the coating area(Points A, B, C and D are shown in the figure); (**b**) Scanning points in the bonding area between the coating and substrate(Points E, F and G are shown in the figure); (**c**) spectrogram A elemental distribution; and (**d**) spectrogram E elemental distribution.

Figure 8a,b show the SEM morphology of the LDED Ni60/60% WC coatings deposited at a laser power of 1200 W. There are undissolved white WC particles (area A), lumpy precipitates around the unmelted WC particles (area B), black streak-like precipitates (area C), the matrix phase (area D), and a fine lumpy microstructure (area E) in the coating. Table 3 shows the results of the energy dispersion mapping analysis of the different zones. It can be observed that the coating mainly consists of white particles of WC in area A, grayish-white W-enriched carbides precipitated in area B, gray Cr-enriched carbides striated in area C, black bonded metal Ni-based solid solution dendrites in area D, and a Cr- and W-enriched fine microstructure in area E [19]. Figure 8c shows the content of the elemental distribution of spectrum A. Figure 8d shows the elemental distribution of the spectra in area E. The large white particles in spectrum A consist mainly of 98.3% W and trace amounts of C. The grey areas in spectra C and E contain Ni, Fe, Cr, and FeNi_3_ phases, which are judged to be present. The grey areas in spectra C and E mainly contain Ni, Fe, Cr, and W, which are suspected to be the Cr_23_C_6_ and FeNi_3_ phases. Since the WC particles are dissolved in the Ni60 matrix, W can also be seen and detected. The black region in spectrum D is mainly in a bonded metal region, so it basically consists of most Ni elements and a small portion of Fe elements, which can be deduced to be the FeNi_3_ phase. Spectrum F shows the bonding region between the coating and the substrate, where the Fe element content increases to 29.8%. Spectrum G is the metal region of the substrate near the bonding region, which is basically dominated by Fe elements. An analysis of the spectral results shows that the proportion of Fe increases with a decreasing distance between the coating surface and the stainless steel substrate. This indicates that, after melting at a high laser power, the Fe elements in the stainless steel substrate gradually enter the coating with the flow of the molten pool and then form FeNi_3_ and FeCr with Ni and Cr in the coating at a high temperature. The main component of the energy spectrum F is Cr, so it is inferred that this position is dominated by Cr_23_C_6_ and FeCr. The presence of significant amounts of Fe and phases associated with elemental Fe in the portion of the coating bonded to the substrate suggests that the Ni60/60% WC coating has a good metallurgical bond with the 316L stainless steel substrate, which is consistent with the results reported in reference [20].

The increased microhardness of the coatings is mainly attributed to the homogeneous diffusion of reinforcements in the matrix playing a diffusion strengthening role and the good bonding strengthening between the reinforcements and the matrix, as well as the bonding strength between the substrate and the coatings. Compared with the substrate 316L stainless steel, Ni60/60% WC composite coatings have a high content of eutectic carbide hard phases, which play a pinning role in the coating microstructure, which impedes the dislocation motion in the coating microstructure. The microhardness value of the coating surface changes as the laser power increases, first increasing and then decreasing. Interestingly, the microhardness of the substrate–coating bonding area changed smoothly, which is a good indication of the diffusion of the atoms between the coating and the substrate, exhibiting excellent metallurgical bonding. At a laser power of 1000 W, the average microhardness of the coating surface reached the maximum value of 937.47 HV. This indicates that smaller laser energy input conditions are unfavorable for the melting of high-melting WC particles, which leads to the emergence of more defects (cracks and pores) in the composite coatings, whereas a larger laser energy input produces a larger number of defects (cracks and pores).

For the friction performance tests, the decrease in the friction coefficient can be attributed to the fact that some of the reinforced particles adhered to the friction partner and surface and cannot be effectively removed, acting as a lubricating medium between the friction balls and the surface and resulting in a decrease in the coefficient of friction. The friction factor curve fluctuates due to the presence of carbides in the coating and the increase in adhesive wear. The lubrication effect of the coating is enhanced at a certain power. In addition, the coating enters the stable wear stage faster at the powers of 1000 W and 1200 W, indicating that the coating has better wear resistance. It can be safely concluded that an appropriate laser power allows the powders to achieve complete melting and then solidification in a shorter time, thereby increasing its density and bonding strength. This helps reduce initial wear and enables the coating to reach a stable wear stage more quickly. However, excessively high power may lead to overheating, making it more easy to wear.

**Figure 9:**
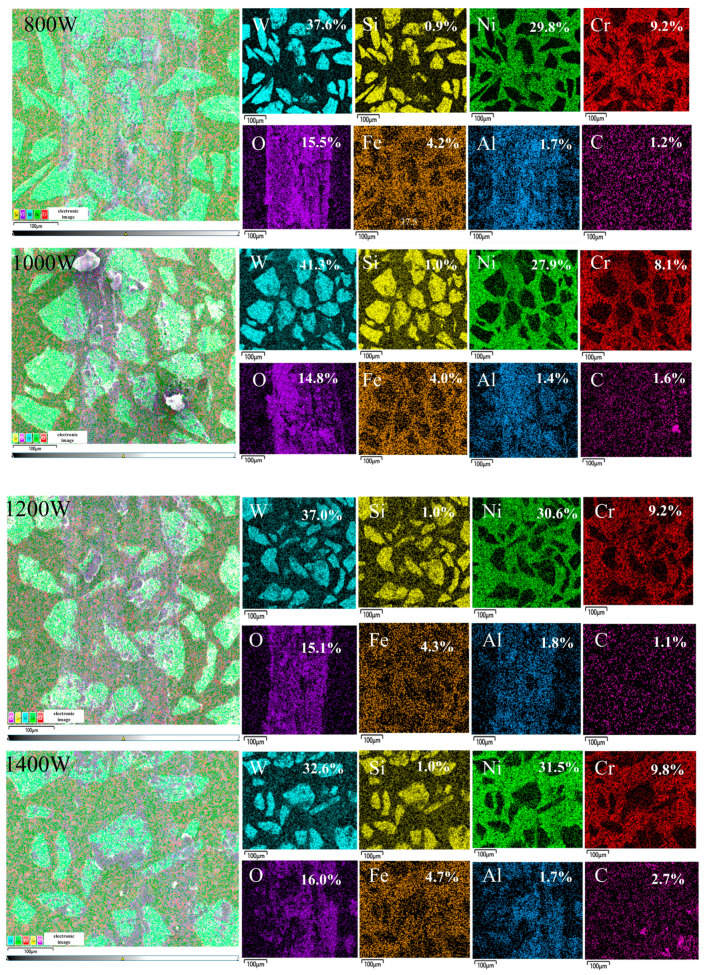
Distribution of EDS elements on friction–wear surfaces of the LDED Ni60/60% WC composite deposited at different powers.

To further investigate the effect of laser power on the friction and wear properties of Ni60/60% WC coatings, the chemical element distribution of the wear marks was analyzed by EDS surface scanning, and Figure 9 shows the EDS plots of the wear specimen surface at different laser powers. From the color distribution of the EDS plot, it can be inferred that the grey particles are carbide and WC particles and the flaking debris on the wear surface are oxides. It can also be observed that oxidative wear has occurred on the wear surface [21]. Moreover, the mass fraction of O at the abrasion marks was significantly higher than that of the unworn coating, indicating that the coating was oxidized during the friction process, and the exposed abraded surfaces reacted with O to produce an oxidation reaction, which exacerbated the friction process. The presence of an oxide layer can make the material more fragile and more prone to crack and peel, which can affect wear performance, especially in high-load rotating friction pairs or high-wear conditions. The results of the EDS surface scanning analysis show that the gradual increase in the laser power results in a more uniform distribution of elements on the wear surface, whereas, at a laser power of 1400 W, the content of O increases and the adhesive wear is intensified, which is consistent with the results of the amount of wear in Figure 6b.

The Al elements in the scanned images are derived from the adhesion of the friction pair. The distribution of Al elements in the worn area of the EDS image is significantly higher than in the unworn area, which is due to the deformation of the friction pair material in the contact region during continuous friction, resulting in local metal adhesion. During subsequent friction, the adhesion of the coating is broken, causing it to peel away from the surface of the metal substrate. This wear mechanism is known as adhesive wear. Moreover, grooves are shown on the surface of the coating, indicating that it is mainly affected by micro-cutting action. Thus, the main wear mechanisms on the coating surface are adhesive and abrasive wear. The presence of a significant amount of hard phases (e.g., WC) in the coating can reduce adhesive wear, as these hard phases help to decrease the material’s tendency to adhere. Additionally, the presence of hard phases improves the microhardness of the coating, thereby enhancing its resistance to abrasive particle erosion.

## 5. Conclusions

In this paper, Ni60/60% WC composite coatings with a good surface roughness and high mechanical properties were successfully prepared on a 316L stainless steel substrate using LDED technology. The effects of laser power on the microstructural evolution and mechanical properties of the Ni60/60% WC composite coating were investigated. The conclusions obtained are as follows:

1. The laser power has a significant effect on the energy input, which determines the melting extent of the Ni60 phases around the WC particles and the bonding strength between the reinforcements and the matrix, as well as the bonding strength between the substrate and the coatings.

2. The variation in laser power significantly affects the microhardness of the coating surface. The average hardness of the coated surface increased to a maximum of 937.47 HV at a laser power of 1000 W due to the increased densification of the deposited coatings, and decreased to 867.71 HV at a laser power of 1400 W due to grain coarsening under a high energy input.

3. The changes in laser power significantly impact the frictional performance of the coating surface. The average coefficient of friction of the coatings decreased gradually to 0.383 at 1000 W, showing a minimum wear of 0.00013 mm^2^ at 1200 W. The main wear mechanisms on the coated surfaces were adhesive wear and abrasive wear. Moreover, the coatings deposited at 1200 W exhibited better forming quality and wear resistance.

This work suggests that the processing parameters during LDED can be optimized to prepare Ni60/60% WC wear-resistant coatings with excellent mechanical properties.

Although this study offers valuable insights into process optimization, it has several limitations that warrant further investigation to enhance the research. This current study primarily focuses on the short-term performance of coatings; future work should concentrate on assessing the durability and stability of coatings under long-term use. This can be achieved through extended testing and durability experiments, which will provide a deeper understanding of the long-term application potential of the coatings.

## Figures and Tables

**Table 1:** Chemical compositions of 316L and Ni60 powders.

**316L stainless steel**										
Elements	C	Si	P	S	Cr	Mn	Ni	Mo	N	Fe
Percent (wt%)	0.015	0.450	0.027	0.005	16.080	1.150	10.090	2.030	0.048	Bal.
**Ni60 powders**										
Elements	C	Si	P	S	Cr	B		Fe		Ni
Percent (wt%)	0.80~1.20	3.50~4.00	0.02	0.02	14.00~16.00	3.00~3.50		14.00~15.00		Bal.

**Table 2:** Process parameters used in the LDED process.

Laser Power (W)	Scanning Speed (m/s)	Powder Feed Rate (g/min)
800	0.010	10
1000	0.010	10
1200	0.010	10
1400	0.010	10

**Table 3:** EDS test results of typical areas of the LDED Ni60/60% WC composite.

	W	C	Ni	Fe	Cr	Si
A	98.3	1.7				
B	52.8	1.2	22.9	2.8	16.7	3.6
C	16.7	1.1	20.6	10.9	50.6	0.1
D			82.3	7.9	4.3	5.5
E	32.7		13.1	7.9	46.3	
F	7.0	1.0	9.0	29.8	53.2	
G		0.3	10.3	71.4	18.0	

## Data Availability

The original contributions presented in the study are included in the article, further inquiries can be directed to the corresponding authors.
